# Rehabilitation Outcomes in Patients Who Regained Consciousness

**DOI:** 10.3390/neurolint18080158

**Published:** 2026-08-21

**Authors:** Elena Aidinoff, Almothana Khatib, Anna Oksamitni, Ilana Gelernter, Lilach Front, Amiram Catz, Sharon Shaklai, Marina Motin, Corinne Serfaty, Deborah Rubin-Asher

**Affiliations:** 1 The Intensive Care for Consciousness Rehabilitation Department, The Departments of Spinal Rehabilitation, Pediatric Rehabilitation, Stroke Rehabilitation, and Brain Injury Rehabilitation, Loewenstein Rehabilitation Medical Center, 278 Achuza St., POB 3, Raanana 4355840, Israel; aidinofe@clalit.org.il (E.A.); motanakhateeb@gmail.com (A.K.); annado@clalit.org.il (A.O.); lilachf@clalit.org.il (L.F.); sharons5@clalit.org.il (S.S.); mottin@clalit.org.il (M.M.); corinnes@clalit.org.il (C.S.); dasher@clalit.org.il (D.R.-A.); 2 The Department of Rehabilitation, The School of Mathematics, Tel Aviv University, Tel Aviv 6139001, Israel; stat61@tauex.tau.ac.il

**Keywords:** consciousness disorders, rehabilitation, brain injuries, cognition, functional status

## Abstract

**Background/Objectives**: Patients with prolonged disorder of consciousness (PDOC) who regained consciousness in intensive care for consciousness rehabilitation (ICCR) were referred to further brain rehabilitation (FBR). The current retrospective cohort study evaluated the functional and cognitive outcomes of FBR. **Methods**: We assessed outcomes in 258 PDOC patients during FBR and used multivariate regression analyses to examine the influence of demographic and clinical characteristics on outcomes. **Results**: During FBR, Functional Independence Measure (FIM) motor, cognitive, and total scores improved from 21 ± 11, 11 ± 10, and 32 ± 15 to 43 ± 26, 18 ± 7, and 61 ± 32, respectively (*p* < 0.001). Dynamic Lowenstein Occupational Therapy Cognitive Assessment (DLOTCA) total scores increased from 57 ± 29 to 82 ± 36, and the average 6-min-walk (6MW) distances improved from 295 to 374 m (*p* < 0.001). Higher discharge FIM and DLOTCA scores were associated with younger age, traumatic etiology of the brain injury, shorter time to regaining consciousness, and higher FIM and DLOTCA scores at admission to FBR (*p* < 0.05). **Conclusions**: We observed functional and cognitive improvement in the examined patients. The improvement was associated with factors that may mainly reflect the severity of brain damage. The findings support continuing rehabilitation before and after emerging from PDOC.

## 1. Introduction

Traumatic and non-traumatic brain injuries may result in prolonged disorders of consciousness (PDOC). PDOC include unresponsive wakefulness syndrome (UWS), which is characterized by sleep–wake cycles and reflexive and spontaneous behaviors, without evidence of awareness, and the minimally conscious state (MCS), in which there is some evidence of self or environmental awareness [1]. Characterization of PDOC is performed by bedside examination and can be assisted by electroencephalography (EEG) techniques and neuroimaging [2].

Recovery from UWS or MCS to a conscious state characterized by functional interactive communication or by functional use of objects occurs mainly during the first months following brain injury [1,3]. Over the last decades, consciousness recovery rates increased [4,5], and consciousness recovery was reported even after a longer time [6,7]. Consciousness recovery was found to be related to characteristics of brain injury, length of PDOC, and EEG response to eye opening [8,9]. In some cases, imaging (MRI) or electrophysiology (EEG) may predict consciousness recovery [5]. Eye tracking responses may also be useful in the evaluation of patients with PDOC [10,11,12]. Recently, researchers examined the effect of emerging rehabilitation technology such as non-invasive brain stimulation techniques on consciousness recovery [13]. Machine learning and deep learning models may assist the diagnosis and prognosis of PDOC patients, identifying factors that influence recovery, and artificial intelligence may assist in creating computer-generated multi-sensory experiences [14,15]. Based on evidence of the efficacy of multidisciplinary approaches for PDOC management, international rehabilitation guidelines recommended that neuro-rehabilitation of patients with prolonged PDOC include teamwork, preferably in a specialized unit. Guidelines recommend that PDOC rehabilitation should focus on the management of the primary medical condition and complications, routine evaluations based on standardized evaluation tools, which can be assisted by monitoring behavior, electroencephalography (EEG), and neuroimaging, as well as on the assistance required for everyday body functions, family support, and decision making for further treatment [1,2]. Recommendations also included reference to individualized rehabilitation planning, family-centered care, community reintegration, and steps aimed to improve health-related quality of life [16,17,18].

Improvement in consciousness recovery, however, did not eliminate brain impairments after this recovery. Patients who regained consciousness still suffer from cognitive and functional impairments [5,19], which have a negative effect on life satisfaction and the cost of care [20,21].

Therefore, after regaining consciousness in an intensive care unit, in some facilities, patients undergo further rehabilitation at the same unit [22], and in certain facilities, at a different one [23,24]. At the facility where the study presented in this article was conducted, after consciousness rehabilitation and regaining consciousness in an intensive care for consciousness rehabilitation (ICCR) department, patients may undergo further brain rehabilitation (FBR) in another specialized rehabilitation department, as detailed in the Methods section. At this stage, neuroplasticity may be enhanced to improve cognitive and motor outcomes by traditional methods, as well as by virtual reality and brain–computer interface technologies [15].

Several publications reported on functional improvement in patients after PDOC. The improvement was more noticeable in patients of younger age, with milder brain injuries, MCS rather than UWS, fewer medical complications, shorter PDOC period, and male sex [19,22,25,26]. Information on the functional and cognitive outcomes of patients with UWS or MCS who underwent FBR after ICCR and consciousness recovery remains limited; however, in most cases, these were investigated in small groups, and none of the studies focused on the outcomes of FBR itself, after ICCR.

To consolidate this information and fill the knowledge gap regarding the outcomes of FBR itself, the present study assessed functional and cognitive outcomes and the factors affecting them during FBR, in a relatively large cohort of PDOC patients who regained consciousness after ICCR.

## 2. Materials and Methods

### 2.1. Study Population

In a retrospective single-center cohort study, we screened the medical records of patients with PDOC, following traumatic and non-traumatic brain injury (TBI and NTBI, respectively), who were referred to an ICCR department in a rehabilitation medical center (RMC) between 2010 and 2021. Inclusion criteria were admission to ICCR, age 16 and above, a clinical diagnosis of UWS or MCS at admission to ICCR, admission to FBR after consciousness recovery, and stay in FBR at one of several departments of the RMC for at least 28 days (expecting to observe rehabilitation effects within 28 days). Exclusion criteria were missing data regarding consciousness state before FBR, no data on FBR outcomes, and nonconformity of the clinical UWS or MCS diagnosis with that based on the Coma Recovery Scale Revised (CRS-R) scores (except for some cases where intact consciousness was clinically verified despite being misclassified based on the scale because of severe motor or language impairment) [27].

The ICCR program included medical care to prevent and treat medical problems, focusing on complications of brain damage, including those of the respiratory and urinary systems, swallowing impairments, pressure sores, spasticity, contractures, intracranial pressure or infection, and wound infection. To optimize the patients’ physical condition, the staff strictly adjusted fluids and electrolytes and conducted bladder and bowel management, close clinical follow-up, neurosurgical consultations, and repeated radiological assessments, as required. For optimal functioning, in addition to medication and nursing, the care included physical measures, adaptive measures (such as placing), and aids (such as splints, adapted wheelchairs, and tilt tables). To enhance arousal and recovery of awareness and to improve communication, patients received medication and environmental stimulation during physiotherapy and occupational therapy, Snoezelen, and music therapy, among others. Alternative communication measures were used when relevant. Close surveillance was conducted to detect new opportunities for communication. When ongoing medical follow-up was indicated, patients were referred to neurosurgery for interventions such as shunt insertion or surgical control of infection. These activities were carried out by a multidisciplinary team consisting of physiatrists, nursing staff, occupational therapists, physiotherapists, speech language pathologists, a psychologist, social workers, a dietitian, and a music therapist, who cared for the patients and supported and advised their families.

At FBR, physiatrists coordinated and directed multidisciplinary teams similar to those of the ICCR, to continue optimizing the medical care, focusing on prevention and treatment of ongoing medical problems and late complications of the brain injury, and on achieving the maximum ability realization assessed as possible considering the patients’ medical condition. Training focused on physical, cognitive, and language functioning at levels that are higher than those addressed at the ICCR, with more attention and intensity devoted to higher physical functioning, cognition, speech, and the patients’ psychological condition.

### 2.2. Procedure

Investigators who were not involved in the care of the included patients reviewed the medical records of the screened patients and retrospectively collected demographic and clinical data. Data from the ICCR records included CRS-R scores, the consciousness state diagnosis based on them, and the Loewenstein Communication Scale (LCS) scores assessing the level of communicativeness [28,29]. CRS-R scores were formally recorded only since 2018. When CRS-R scores were missing in the medical records, they were determined retrospectively by an occupational therapist with over 30 years of experience and training in CRS-R administration, based on descriptions that the ICCR staff had recorded. Data from the FBR records included the scores at discharge from FBR and the difference between discharge and admission scores of the Functional Independence Measure (FIM) motor, cognitive, and total scores (range 18–126; higher scores representing better functioning) [30], and of the Dynamic Loewenstein Occupational Therapy Cognitive Assessment (DLOTCA) domain and total scores (maximum possible score = 138; higher scores representing better functioning). FIM is the most widely used tool for assessing function in individuals with a disability and has undergone extensive validation and reliability testing [31,32]. DLOTCA is useful in detecting cognitive deficits and was found reliable and valid [33,34]. The FBR data also included the walking distance in the 6-min walk test (6MW, reported range for healthy people 380–782 m), assessed for patients who could walk, when they started walking unassisted, and at discharge [35]. Data were entered into an Excel file for analysis.The statistical analysis was performed using the Statistical Package for Social Sciences (SPSS) version 31.0 for Windows (IBM Inc. Armonk, NT, USA).

### 2.3. Ethical Considerations

The Institutional Review Board of Loewenstein Rehabilitation Medical Center (Protocol code 0014-20-LOE, approved 8 July 2020) approved the study and waived the requirement to obtain informed consent from the patients because the study was retrospective and the investigators anonymized the data before analysis.

### 2.4. Statistical Analysis

The patients included in the analysis and those who qualified for inclusion but were excluded from the study were compared using independent sample T- and chi-square tests for continuous and discrete variables, respectively. The differences between admission and discharge outcomes were evaluated using paired sample *t*-tests, and the minimal clinically important difference (MCID) was calculated as the standard deviation (SD) at admission × 0.5 [36]. Relationships between various factors and outcomes at discharge from FBR, and between various factors and the difference from admission to discharge, were evaluated by bivariate analyses, using independent sample *t*-tests and Pearson correlations with Bonferroni corrections for multiple comparisons, followed by multivariate linear regressions, to assess independent relationships with outcomes. We entered the variables that were significant in the bivariate analyses into the multiple regression analyses. The DLOTCA admission score, however, which was often missing, was omitted from the regression analyses if otherwise the number of patients would have been severely reduced. We used bootstraps (1000 repetitions) to generate robust estimates. We evaluated multicollinearity with variance inflation factors (VIF), looking for values above 5. Residual diagnostics were evaluated.

## 3. Results

### 3.1. Patients

**Figure 1:**
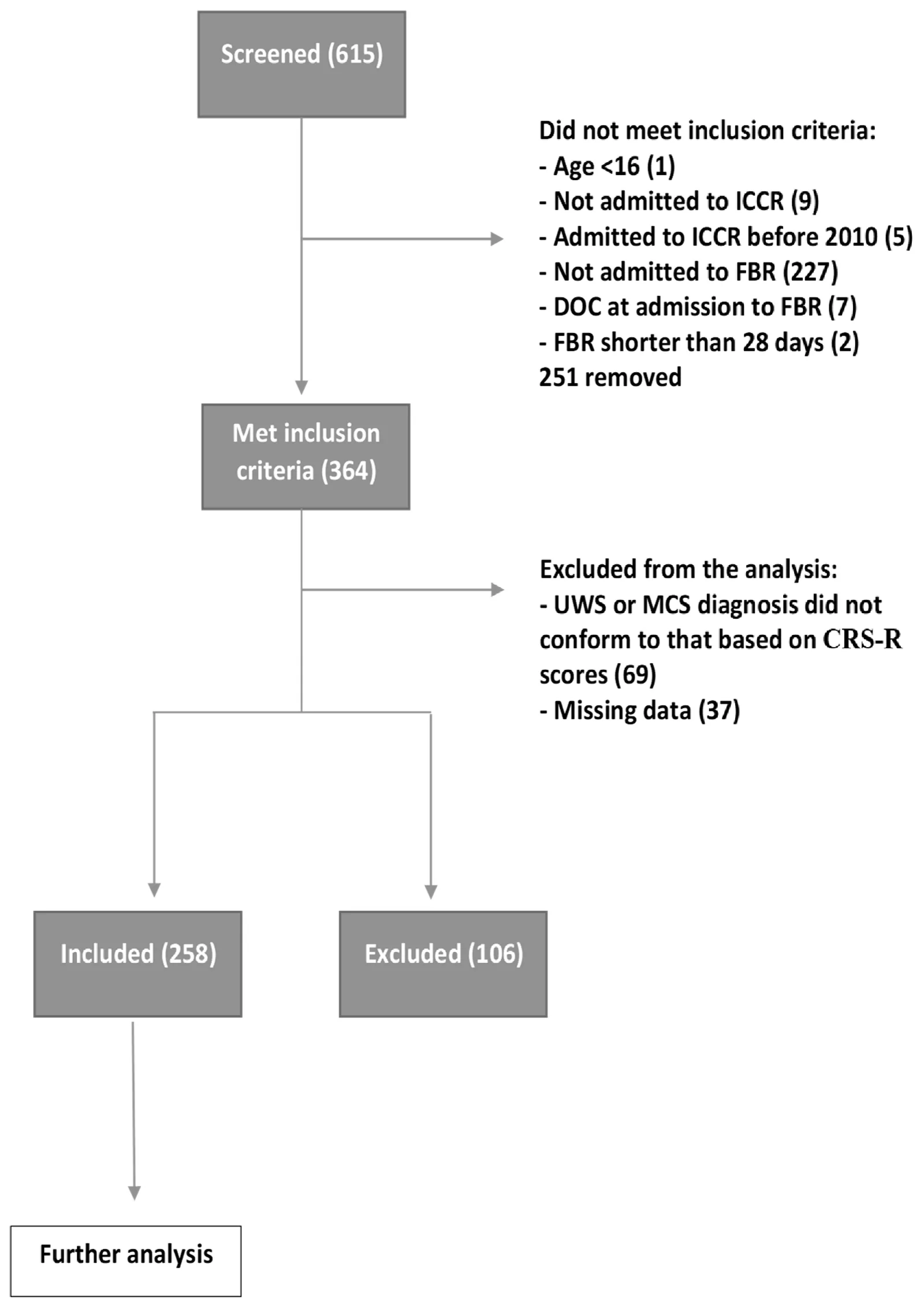
Flow chart of inclusion and exclusion criteria.

Of 615 patients screened for the study, 364 met the inclusion criteria, and 106 of them were excluded. Sixty-nine were excluded because retrospective CRS-R assessment showed that they were conscious on admission to ICCR and did not meet the inclusion criteria. Eleven patients were not excluded despite low CRS-R scores at discharge from ICCR because clinical judgement indicated that the scale misclassified them owing to severe aphasia or motor impairment. Of the remaining 295 who met the CRS-R inclusion criteria, 37 were excluded for missing data, leaving 258 patients (87%) who participated in the analysis (Figure 1).

All 258 patients included in the study had been in PDOC for 28 days or more, except one who recovered consciousness after 25 days in MCS. All of them had available CRS-R scores, and more than 92% had LCS scores.

Demographic and clinical data of the 258 included patients are presented in Table 1.

The 258 patients included in the analysis had a lower consciousness level at admission to ICCR than the 37 patients who had PDOC at admission to ICCR but were excluded (41% UWS and 59% MCS in the 258 included patients, and 23% UWS and 77% MCS in the 37 excluded, *p* < 0.001). The 37 excluded patients and the 258 included in the analysis did not significantly differ in age (46 ± 19 vs. 45 ± 18, *p* > 0.3), length of stay (LOS) in ICCR (63 ± 86 vs. 70 ± 48 days, *p* > 0.2), percentage of men (76% vs. 71%, *p* > 0.3), or etiology of brain damage (TBI in 61% and 63% of the excluded and included patients, respectively, *p* > 0.8).

CRS-R evaluations were performed retrospectively for 148 (57%) of the patients at admission to the ICCR and for 180 (69%) at discharge. The CRS-R evaluations and the clinical diagnosis of consciousness were in total agreement regarding the eligibility for inclusion in the study in 247 of 327 patients with available CRS-R data (76%). The prospective and the retrospective CRS-R evaluations did not significantly differ in the score value (8.7, SD = 4.1 and 9.2, SD = 4.8, respectively) or in the UWS/MCS ratio (38%/62%, and 45%/55%, respectively) (*p* > 0.1).

### 3.2. Outcomes

FIM scores at admission to FBR and at discharge were available for all patients but one. DLOTCA total scores were available for 153 patients (59%) at admission, and 117 (45%) at discharge, and 6MW data for 52 patients (20%) at start walking without assistance, and 78 (30%) at discharge. The percentage of patients with TBI of those with available DLOTCA scores was lower than that of those with missing DLOTCA data (57% and 70%, respectively, *p* = 0.037).

The scores of FIM, DLOTCA, and the 6MW distance, at start walking without assistance and at discharge from FBR, are presented in Table 2. Differences between admission and discharge scores were significant and exceeded the MCID. At discharge, 47 patients, 18% of the patients who regained consciousness in ICCR, achieved a total FIM score ≥ 100, and 15 (5.8%) achieved a score ≥ 5 on all FIM subscales. The mean total FIM and DLOTCA scores at admission to FBR were 25% and 41% of their maximum, and at discharge, 48% and 59%, respectively. The mean 6MW distances at admission and discharge were 50% and 64% of the average value of the reported range for healthy people, respectively. FIM and DLOTCA total and domain scores, as well as 6MW distances, increased significantly during FBR stay (*p* < 0.001). Two patients (0.8%) died during FBR, and 7 (2.7%) within about one year after being transferred from FBR, owing to clinical deterioration.

### 3.3. Particularly Remarkable Functional Achievements at FBR

One patient achieved scores of 6 or 7 on all FIM subscales, which means that after FBR, he did not require any assistance in basic daily activities. The other 14 patients who achieved scores ≥ 5 on all FIM subscales became independent in daily activities after FBR, at least with environmental adaptation and supervision or with guidance and encouragement. They needed supervision or guidance mainly in tasks of memory, problem solving, stair-climbing, washing, and showering. Of the 14, 6 were also independent in tasks of extended daily activities.

After discharge from FBR, a 17-year-old patient achieved independent ambulation and driving; a 19-year-old patient had intact memory, managed a daily schedule independently, conducted coherent conversations, and showed learning and planning ability; a 33-year-old patient was independent in all basic and extended daily activities; a 41-year-old patient had functional speech, good writing ability, and ability to walk any distance; and a 62-year-old patient had no cognitive deficit.

### 3.4. Relationship Between Outcomes and Patient Characteristics

Relationships between outcomes and patient characteristics are presented in Table 3, Table 4, Table 5, Table 6, Table 7, Table 8, Table 9, Table 10, Table 11 and Table 12. Many of these relationships are similar for most outcomes, but outcomes differ in some.

Among the associations that were examined only in bivariate analyses, the 6MW distance at discharge from FBR was found to be associated with LOS in ICCR and with FIM score at admission to FBR (Table 6 and Table 9). The LOS in FBR was negatively correlated with motor and total FIM scores and with DLOTCA scores at FBR admission (r = −0.190, −0.166 and −0.304, respectively, *p* < 0.01) and was not significantly correlated with FIM cognitive score at admission to FBR or with CRS-R score at admission to ICCR.

Relationships between outcomes and patient characteristics, after controlling for factors that were associated with the outcomes in the bivariate analysis, are presented in Table 13 and Table 14. The functional outcome FIM motor at discharge from FBR and its gain during FBR were found independently related to younger age, TBI, and time from injury to admission to FBR. At discharge, they were independently related to FIM score at admission to FBR, and the gain was also independently related to a diagnosis of hydrocephalus. The gain of cognitive outcomes, FIM cognitive and DLOTCA, was independently associated with LOS in FBR. Discharge FIM cognitive was independently associated with younger age, shorter LOS in ICCR, lack of hydrocephalus diagnosis, and FIM total at admission to FBR, and its gain with shorter LOS in ICCR. Discharge DLOTCA was also independently associated with its score at admission to FBR. Sex, consciousness state (UWS or MCS) at admission to ICCR, CRS-R and LCS scores, procedures, and most complications were not found to be independently related to outcomes. No excessive multicollinearity was observed. Bootstrapping analyses were consistent with the outcomes. The assumptions for linear regression were verified.

## 4. Discussion

This is the first report that focuses on outcomes of FBR itself after ICCR. It describes functional and cognitive achievements in a large group of patients with PDOC who regained consciousness. Participants were patients with TBI and NTBI who underwent ICCR, followed by FBR, after consciousness recovery.

A significant positive difference was found in all outcomes, above MCID, between admission and discharge from FBR scores (Table 2). After FBR, the mean FIM total score was 61 (SD = 32), and in 18% of patients, it was ≥100, reflecting independence that allowed discharge to the community [37]. The included patients achieved reasonable functional, motor, and cognitive outcomes, reaching, on average, 48% and 59% of the maximum possible FIM and DLOTCA scores and 64% of the average 6MW distances of the range reported for healthy people. We found improvement during FBR in these outcome measures in 44–100% of the patients. A non-negligible portion of them (5.8%) needed no assistance in any daily task. Temporal trends in clinical practice did not affect outcome, which did not significantly differ between 2010–2015 and 2016–2021 (Table 1 and Table 4).

Although most of the patients who regained consciousness after PDOC did not reach complete independence in daily activities, the improvement during FBR can be considered significant. This is because many achieved a remarkable functional state, ranging from independent functioning in one or several important tasks to complete independence. These achievements were good by comparison with what was expected based on other studies [21,22,26,38,39], in the highly dependent population of patients after ICCR, and a non-negligible portion of the patients achieved very high functioning.

FIM gain and discharge FIM scores in this study were comparable to those found in most previous studies of patients who regained consciousness after a brain injury and intensive rehabilitation programs or better [21,22,26,38,39]. Functional outcomes were better, however, in studies that included only TBI patients [40,41]. This is consistent with the relationship found between better outcomes in TBI than in NTBI in this study, as well as in previous studies, which may indicate that the studied TBIs were less severe than the NTBIs [25,39,40,41,42].

Better functional and cognitive outcomes at discharge from FBR were also independently related in this study to younger age, shorter time from brain injury onset to regaining consciousness and admission to FBR, higher FIM or DLOTCA scores at admission to FBR, and lack of diagnosis of hydrocephalus during rehabilitation (Table 13). Improvement of outcomes during FBR was independently related to the same factors, except FIM scores at admission to FBR, which were negatively associated with outcomes. In addition, we found a positive relationship between outcome improvement and LOS in FBR (Table 14). The effects of age, which may be associated with lifetime accumulation of minute brain damage, time from brain injury onset to regaining consciousness, and hydrocephalus, probably reflect a lower potential for recovery owing to more severe brain injury. Patients who had better functional or cognitive performance at admission to FBR showed better outcomes at discharge, probably because of less severe injuries or better recovery at ICCR. The inverse relationship between admission scores and improvement may reflect regression to the mean, but it probably primarily reflects a clinical ceiling effect, as patients with higher FIM scores at admission to FBR showed higher scores at discharge, the functioning of about 90% of the patients improved from admission to discharge, and it is likely that improvement halted as patients approached their rehabilitative potential. The relationship between longer stay in FBR and improvement in outcomes may suggest that patients whose function did not improve were discharged from rehabilitation earlier. But the fact that LOS in FBR was shorter in patients with better admission performance does not support this possibility. Greater improvement can be attributed, therefore, to the length of stay in rehabilitation and probably to the effect of rehabilitation itself.

Previous studies have also shown that younger age [39,43,44], TBI [25,39], less severe brain damage [19,22,25,45,46], shorter time to regaining consciousness [19,25], no hydrocephalus [26], and longer rehabilitation [45] have a positive effect on motor and functional outcomes in patients with brain injuries. Unlike other studies [22,25], the present one found no independent relationship between being in UWS or MCS at admission to ICCR and functional outcomes after regaining consciousness. The reason for this difference is not clear but may be explained by the differences between the populations that participated in the various studies. Other studies that have examined this relationship included the entire ICCR population with UWS or MCS, whereas the present study included only those selected for FBR. It makes sense that the UWS patients who regained consciousness and were selected for FBR were those in UWS with milder brain damage, so the severity of their brain damage was close to that of the patients in MCS.

Short-term mortality after admission to FBR (3.5%) was much lower than that found in our previous study in patients admitted to the same ICCR department with UWS (12–27%) [4]. This finding is not surprising, as the patients in FBR were ICCR patients who regained consciousness, and it is consistent with other findings showing that consciousness recovery reduced the risk of mortality [47].

The functional and cognitive outcomes of the patients in this study, as well as in smaller groups with PDOC who underwent special programs similar to the ICCR and FBR after consciousness recovery [22,25,26], attest to the importance of ICCR and FBR for achieving favorable outcomes after PDOC. Achievements reported in this and other relatively recent studies reduce doubts voiced in previous years about the ethical justification of operating intensive care units for these patients, citing the high cost and the suffering of the patients’ relatives [39,48], and demonstrate the need to refer PDOC patients to ICCR and those who regain consciousness to FBR. Ethical justification does not depend only on clinically prominent efficacy or cost-effectiveness. The fact that intensive rehabilitation enables patients to attain functional milestones is of paramount importance for both their families and society [23,49]. Therefore, society must not forgo the opportunity to facilitate such life-changing recovery for these individuals.

### 4.1. Study Limitations

A limitation of the study is that it did not address complications that occurred before rehabilitation and could affect outcomes. This limits the generalizability of the conclusions to patients with a similar rate of pre-rehabilitation complications. Another limitation is that the study focused only on basic functional outcomes and did not assess extended activities, psychological adjustment, perception of health, or quality of life. Most of these outcomes, however, are important mainly in the community, after discharge from inpatient rehabilitation. An additional limitation is the retrospective single-center design, which can limit external validity; the data consists of past records that may be incomplete, the findings lack geographic diversity, and some specific rehabilitation modalities are needed to generalize to other healthcare settings. The absence of a comparison group, no long-term follow-up, and the possible effects of missing data are further limitations. The values of R^2^ in the multivariate linear regressions, which indicate that the patient characteristics found to be related to the outcomes explain only a low to medium portion of the variation in outcomes, limit the predictive value of these characteristics. These limitations, however, do not derogate from the functional and cognitive achievements during FBR after PDOC demonstrated in the study. The possible effect on generalizability of selection bias related to the lower consciousness state at admission to ICCR in the patients included in the analysis, when compared with the qualifying excluded, may also be considered a limitation. But no other significant differences were found between these two groups, and the difference we found indicates that the functional achievements at FBR may be even better than those presented, supporting the conclusions of the study.

### 4.2. Future Research

Future research may include additional paths for exploration, such as long-term follow-up post-discharge, community participation, vocational reentry, caregiver results/outcomes, and the potential contributions of new rehabilitation technologies like virtual reality, robotics, brain–computer interface, and AI-supported rehabilitation [15,50].

## 5. Conclusions

FBR for patients with PDOC whose consciousness recovered after ICCR achieved functional and cognitive improvement, which can be considered significant in patients after ICCR. Younger age, traumatic etiology, not having hydrocephalus, shorter time to regaining consciousness, and better function at admission to FBR were associated with better functional or cognitive outcomes at the end of rehabilitation. The main outcomes support rehabilitation for these patients before and after consciousness recovery.

## Figures and Tables

**Table 1:** Patient data.

Variable	Value	Range
Number	258	
Admitted to FBR between 2010 and 2015, n (%)	85 (33)	
Admitted to FBR between 2016 and 2021, n (%)	173 (67)	
Age (years), mean (SD)	45 (18)	16–87
Sex, n (%)		
Male	184 (71)	
Female	74 (29)	
Education, n (%)		
None, primary, secondary	125 (74)	
Beyond secondary	67 (26)	
Etiology, n (%)		
TBI	162 (63)	
NTBI	96 (37)	
Time from injury to ICCR (days), mean (SD)	45 (35)	9–244
Consciousness state at admission, n (%)		
UWS	102 (40)	
MCS	156 (60)	
Length of stay in ICCR (days), mean (SD)	70 (48)	4–303
CRS-R score, mean (SD)		
Admission to ICCR	9.0 (4.5)	0–18
Discharge from ICCR	21.2 (2.4)	12–23
LCS score, mean (SD)		
Admission to ICCR	24 (12)	8–71
Discharge from ICCR	57 (15)	13–98
Procedures in ICCR, n (%)		
Venricoperitoneal shunt insertion	26 (10)	
Cranioplasty	24 (9.3)	
Complications in ICCR, n (%)		
Pneumonia	14 (5.4)	
Hydrocepahlus	52 (20)	
Urinary tract infection	62 (24)	
Pressure ulcer	7 (2.7)	
Venricoperitoneal shunt infection	2 (0.8)	
Length of stay in FBR (days), mean (SD)	150 (93)	
Procedures in FBR, n (%)		
Venricoperitoneal shunt insertion	12 (4.7)	
Cranioplasty	24 (9.3)	
Complications in FBR, n (%)		
Pneumonia	6 (2.3)	
Hydrocepahlus	10 (3.9)	
Urinary tract infection	24 (9.3)	
Pressure ulcer	1 (0.4)	
Venricoperitoneal shunt infection	2 (0.8)	

ICCR = intensive care for consciousness rehabilitation; FBR = further brain rehabilitation; TBI = traumatic brain injury; NTBI = non-traumatic brain injury; SD = standard deviation; UWS = unresponsive wakefulness syndrome; MCS = minimally conscious state; CRS-R = Coma Recovery Scale-Revised; LCS = Loewenstein Communication Scale.

**Table 2:** Outcomes of FBR after discharge from ICCR.

Outcome		N	Mean (SD)	Range	*p*-Value ^a^	Cohen’s d Value
FIM motor	Admission	258	21 (11)	13–75	<0.001	1.091
	Discharge	257	43 (26)	13–90		
	Difference	257	22 (20) *	(−9)–67		
FIM cognitive	Admission	258	11 (10)	5–29	<0.001	1.099
	Discharge	257	18 (7)	5–34		
	Difference	257	6.7 (6.1) *	(−6)–27		
FIM total	Admission	258	32 (15)	18–98	<0.001	1.201
	Discharge	257	61 (32)	18–120		
	Difference	257	29 (24) *	(−11)–88		
DLOTCA orientation	Admission	203	5.7 (5.0)	0–16	<0.001	0.919
	Discharge	135	10.5 (5.3)	0–16		
	Difference	127	4.6 (5.0) *	(−10)–16		
DLOTCA Visual perception	Admission	209	8.4 (3.4)	3–12	<0.001	0.670
	Discharge	139	10.2 (2.9)	10.2 (2.9)		
	Difference	134	1.9 (2.9) *	(−6)–9		
DLOTCA Spatial perception	Admission	207	7.1 (3.5)	2–12	<0.001	0.760
	Discharge	139	8.8 (3.4)	2–12		
	Difference	134	2.1 (2.8 ) *	(−4)–9		
DLOTCA praxis	Admission	205	12.3 (7.7)	2–24	<0.001	0.785
	Discharge	139	17.1 (7.4)	3–24		
	Difference	133	5.2 (6.6) *	(−12)–21		
DLOTCA visuomotor construction	Admission	197	13.3 (7.6)	7–34	<0.001	0.847
	Discharge	138	19.3 (10.3)	7–35		
	Difference	129	6.1 (7.2) *	(−5)–27		
DLOTCA Thinking operations	Admission	189	12.6 (7.1)	8–33	<0.001	0.905
	Discharge	134	18.4 (9.9)	7–37		
	Difference	124	6.0 (6.6) *	(−1)–26		
DLOTCA total	Admission	153	57 (29)	24–127	<0.001	1.053
	Discharge	117	82 (36)	24–136		
	Difference	97	25 (24) *	(−18)–97		
6MW (meters)	Start ^b^	52	295 (123)	65–660	<0.001	1.109
	Discharge	78	374 (189)	30–1000		
	Difference ^c^	47	126 (114) *	(−80)–450		

Abbreviations: FIM = Functional Independence Measure; DLOTCA = Dynamic Lowenstein Occupational Therapy Cognitive Assessment; 6MW = 6-min walk distance. ^a^ For the difference between admission and discharge. ^b^ Start = start walking without assistance. ^c^ Difference = difference between the 6MW distance at discharge from FBR and at start of walking without assistance. * indicates a difference larger than the minimal clinically important difference (MCID), based on 0.5XSD at the baseline.

**Table 3:** Relationship between demographic characteristics and outcomes.

Outcome	Age			Sex					Years of Education		
	r	*p*	*p* (Bonf.)	Male, Mean (SD)	Female, Mean (SD)	*p* ^b^	Cohen’s d Value	*p* (Bonf.)	r	*p*	*p* (Bonf.)
FIM—motor discharge	−0.359	<0.001	<0.05	46 (27)	37 (23)	0.015	0.339	NS	−0.009	>0.8	
FIM—motor gain	−0.322	<0.001	<0.05	24 (21)	19 (19)	>0.05	0.241		0.020	>0.7	
FIM—cognitive discharge	−0.361	<0.001	<0.05	18 (7)	17 (8)	>0.6	0.070		−0.009	>0.8	
FIM—cognitive gain	−0.179	<0.001	<0.05	6.5 (6.1)	7.2 (6.1)	>0.3	−0.126		0.072	>0.2	
FIM—total discharge	−0.377	<0.001	<0.05	64 (33)	54 (21)	0.036	0.291	NS	−0.010	>0.8	
FIM—total gain	−0.315	<0.001	<0.05	30 (25)	26 (22)	>0.2	0.169		0.037	>0.5	
DLOTCA—total discharge	−0.182	0.049	NS	86 (36)	73 (33)	>0.05	0.355		0.108	>0.2	
DLOTCA—total gain	−0.183	>0.05		25 (25)	23 (19)	>0.7	0.087		0.072	>0.4	
6MW discharge	−0.179	>0.1		394 (200)	313 (136)	>0.1	0.437		−0.104	>0.3	
6MW difference ^a^	−0.064	>0.6		127 (119)	123 (101)	>0.9	0.032		0.290	0.048	NS

Abbreviations: r = correlation coefficient; *p* = significance. ^a^ Difference = difference between the 6MW distance at discharge from FBR and at start of walking without assistance. ^b^ for the difference between sexes. *p* (Bonf.) = whether significant (*p* < 0.05) after Bonferroni corrections. NS = non-significant.

**Table 4:** Outcomes for 2010–2015 and 2016–2021.

Outcome	Admission to Rehabilitation			
	2010–2015, Mean (SD)	2016–2021, Mean (SD)	*p* ^b^	Cohen’s d Value
FIM—motor discharge	43 (25)	43 (26)	>0.9	−0.011
FIM—motor gain	23 (21)	22 (20)	>0.5	−0.089
FIM—cognitive discharge	19 (7)	17 (8)	>0.2	−0.144
FIM—cognitive gain	7.3 (5.3)	6.4 (6.4)	>0.2	−0.150
FIM—total discharge	62 (31)	61 (32)	>0.7	−0.043
FIM—total gain	31 (24)	28 (24)	>0.4	−0.111
DLOTCA—total discharge	73 (37)	85 (35)	>0.1	0.354
DLOTCA—total gain	26 (29)	24 (22)	>0.7	−0.065
6MW discharge	371 (202)	375 (185)	>0.9	0.021
6MW difference ^a^	104 (111)	134 (115)	>0.4	0.256

^a^ Difference = difference between the 6MW distance at discharge from FBR and at start of walking without assistance. ^b^ for differences between early and late years of admission.

**Table 5:** Relationship between general clinical characteristics and outcomes.

Outcome	Etiology					Consciousness State at Admission to ICCR				
	TBI, Mean (SD)	NTBI, Mean (SD)	*p* ^c^	Cohen’s d Value	*p* (Bonf.)	UWS, Mean (SD)	MCS, Mean (SD)	*p* ^d^	Cohen’s d Value	*p* (Bonf.)
FIM—motor discharge	51 (26)	30 (20)	<0.001	0.851	<0.05	37 (23)	48 (26)	0.001	0.431	<0.05
FIM—motor gain	28 (21)	13 (16)	<0.001	0.751	<0.05	17 (16)	26 (22)	<0.001	0.507	<0.05
FIM—cog ^a^ discharge	20 (7)	15 (7)	<0.001	0.668	<0.05	17 (7)	19 (8)	0.034	0.272	N.S.
FIM—cog ^a^ gain	7.4 (5.8)	5.4 (6.4)	0.018	0.308	N.S	6.1 (5.9)	7.1 (6.2)	>0.2	0.160	
FIM—total discharge	70 (32)	45 (25)	<0.001	0.853	<0.05	53 (29)	66 (33)	0.001	0.414	<0.05
FIM—total gain	35 (24)	19 (20)	<0.001	0.710	<0.05	22 (20)	33 (26)	<0.001	0.465	<0.05
DLOTCA-total discharge	91(36)	70 (32)	0.002	0.598	<0.05	77 (33)	85 (37)	>0.2	0.224	
DLOTCA—total gain	30 (25)	17.5 (20)	0.010	0.543	N.S.	25 (23)	25 (24)	>0.9	0.007	
6MW discharge	408 (182)	278 (180)	0.007	0.718	N.S.	367 (174)	377 (195)	>0.8	0.053	
6MW difference ^b^	136 (119)	92 (92)	>0.2	0.343		93 (132)	137 (106)	>0.2		

Abbreviations: ^a^ FIM—cog = FIM cognitive. ^b^ Difference = difference between the 6MW distance at discharge from FBR and at start of walking without assistance. ^c^ for TBI vs. NTBI. ^d^ for UWS vs. MCS. *p* (Bonf.) = whether significan—t (*p* < 0.05) after Bonferroni corrections.

**Table 6:** Relationship between additional characteristics and outcomes.

	Time from Injury to ICCR Admission			LOS in ICCR			LOS in FBR		
Outcome	r	*p*	*p* (Bonf.)	r	*p*	*p* (Bonf.)	r	*p*	*p* (Bonf.)
FIM—motor discharge	−0.270	<0.001	<0.05	−0.443	<0.001	<0.05	0.000	1.000	
FIM—motor gain	−0.207	0.001	<0.05	−0.387	<0.001	<0.05	0.107	0.05	N.S.
FIM—cognitive discharge	−0.212	0.001		−0.360	<0.001	<0.05	0.126	0.044	N.S.
FIM—cognitive gain	−0.081	>0.1		−0.192	0.002	<0.05	0.235	<0.001	<0.05
FIM—total discharge	−0.269	<0.001	<0.05	−0.445	<0.001	<0.05	0.030	>0.6	
FIM—total gain	−0.195	0.002	<0.05	−0.375	<0.001	<0.05	0.148	0.017	N.S.
DLOTCA-total discharge	−0.263	0.004	<0.05	−0.327	<0.001	<0.05	−0.046	>0.6	
DLOTCA—total gain	0.023	>0.8		−0.082	>0.4		0.319	0.001	<0.05
6MW discharge	−0.181	>0.1		−0.359	0.001	<0.05	−0.182	>0.1	
6MW difference ^a^	−0.048	>0.7		0.036	>0.8		0.002	>0.9	

Abbreviations: LOS = length of stay. ^a^ Difference = difference between the 6MW distance at discharge from FBR and at start of walking without assistance.

**Table 7:** Relationship between CRS-R scores in ICCR and outcomes.

Outcome	ICCR Admission CRS-R			ICCR Discharge CRS-R		
	r	*p*	*p* (Bonf.)	r	*p*	*p* (Bonf.)
FIM—motor discharge	0.234	<0.001	<0.05	0.308	<0.001	<0.05
FIM—motor gain	0.257	<0.001	<0.05	0.247	<0.001	<0.05
FIM—cognitive discharge	0.184	0.003	<0.05	0.334	<0.001	<0.05
FIM—cognitive gain	0.109	>0.05		0.101	>0.1	
FIM—total discharge	0.233	<0.001	<0.05	0.329	<0.001	<0.05
FIM—total gain	0.241	<0.001	<0.05	0.233	<0.001	<0.05
DLOTCA -total discharge	0.116	>0.2		0.344	<0.001	<0.05
DLOTCA -total gain	0.049	>0.6		0.049	>0.6	
6MW discharge	−0.052	>0.6		−0.051	>0.6	
6MW difference ^a^	0.021	>0.8		0.060	>0.6	

^a^ Difference = difference between the 6MW distance at discharge from FBR and at start of walking without assistance.

**Table 8:** Relationship between LCS scores in ICCR and outcomes.

Outcome	ICCR Admission LCS			ICCR Discharge LCS			LCS Gain		
	r	*p*	*p* (Bonf.)	r	*p*	*p* (Bonf.)	r	*p*	*p* (Bonf.)
FIM—motor discharge	0.200	0.001	<0.05	0.457	<0.001	<0.05	0.318	<0.001	<0.05
FIM—motor gain	0.204	0.001	<0.05	0.351	<0.001	<0.05	0.229	<0.001	<0.05
FIM—cognitive discharge	0.151	0.015	N.S.	0.372	<0.001	<0.05	0.273	<0.001	<0.05
FIM—cognitive gain	0.083	>0.1		0.129	0.047	N.S.	0.061	>0.3	
FIM—total discharge	0.199	0.001	<0.05	0.458	<0.001	<0.05	0.323	<0.001	<0.05
FIM—total gain	0.190	0.002	<0.05	0.328	<0.001	<0.05	0.211	0.001	<0.05
DLOTCA -total discharge	0.059	>0.5		0.427	<0.001	<0.05	0.363	<0.001	<0.05
DLOTCA—total gain	0.103	>0.3		0.015	>0.8		−0.028	>0.8	
6MW discharge	0.050	>0.6		0.327	0.006	N.S.	0.211	>0.05	
6MW difference ^a^	0.145	>0.3		0.240	>0.1		0.052	>0.7	

^a^ Difference = difference between the 6MW distance at discharge from FBR and at start of walking without assistance.

**Table 9:** Relationship between scores at FBR admission and outcomes.

Outcome	FBR Admission FIM			FBR Admission DLOTCA		
	r	*p*	*p* (bonf.)	r	*p*	*p* (bonf.)
FIM—motor discharge	0.690	<0.001	<0.05	0.568	<0.001	<0.05
FIM—motor gain	0.343	<0.001	<0.05	0.467	<0.001	<0.05
FIM—cognitive discharge	0.527	<0.001	<0.05	0.529	<0.001	<0.05
FIM—cognitive gain	−0.084	>0.1		0.199	0.014	N.S.
FIM—total discharge	0.685	<0.001	<0.05	0.587	<0.001	<0.05
FIM—total gain	0.265	<0.001	<0.05	0.443	<0.001	<0.05
DLOTCA—total discharge	0.477	<0.001	<0.05	0.752	<0.001	<0.05
DLOTCA—total gain	0.095	>0.3		−0.157	>0.1	
6MW discharge	0.394	<0.001	<0.05	0.055	>0.7	
6MW difference ^a^	0.245	>0.05		0.117	>0.5	

^a^ Difference = difference between the 6MW distance at discharge from FBR and at start of walking without assistance.

**Table 10:** Relationship between complications at rehabilitation and outcomes (1).

	Hydrocephalus				Pneumonia				
Outcome	No ^a^ Mean (SD)	Yes ^b^ Mean (SD)	*p* ^c^	Cohen’s d Value	*p* (Bonf.)	No ^a^ Mean (SD)	Yes ^b^ Mean (SD)	Cohen’s d Value	*p* ^c^
FIM—motor discharge	46 (26)	33 (22)	<0.001	0.530	<0.05	44 (26)	34 (24)	0.375	>0.1
FIM—motor gain	24 (21)	16 (18)	0.004	0.420	<0.05	23 (20)	17 (22)	0.268	>0.2
FIM—cognitive discharge	19 (7)	15 (7)	<0.001	0.537	<0.05	18 (7)	17 (8)	0.054	>0.8
FIM—cognitive gain	7.1 (6.2)	5.6 (5.7)	>0.05	0.242		6.7 (6.2)	6.8 (5.3)	−0.010	>0.9
FIM—total discharge	65 (32)	48 (27)	<0.001	0.595	<0.05	62 (32)	52 (30)	0.317	>0.1
FIM—total gain	31 (24)	21 (21)	0.005	0.413	<0.05	29 (24)	24 (24)	0.221	>0.3
DLOTCA—total discharge	84 (36)	76 (34)	>0.3	0.212		83 (36)	71 (33)	0.355	>0.3
DLOTCA—total gain	26 (25)	19 (18)	>0.1	0.304		25 (22)	27 (34)	−0.109	>0.1
6MW discharge	386 (195)	294 (124)	>0.1	0.491		366 (167)	700 (424)	−1.832	>0.4
6MW difference ^d^	134 (117)	73 (74)	>0.2	0.504		126 (114)	NA	NA ^e^	NA ^e^

Abbreviations: NA = Not available. ^a^ Outcome when the complication was absent. ^b^ Outcome when the complication was present. ^c^ For differences between presence and absence of the complication. ^d^ Difference = difference between the 6MW distance at discharge from FBR and at start walking without assistance. ^e^ The number of patients in the group was too small for meaningful statistical significance.

**Table 11:** Relationship between complications at ICCR and FBR and outcomes (2).

	Pressure Ulcer					Urinary Tract Infection			
Outcome	No ^a^ Mean (SD)	Yes ^b^ Mean (SD)	*p* ^c^	Cohen’s d Value	*p* (Bonf.)	No ^b^ Mean (SD)	Yes ^c^ Mean (SD)	*p* ^c^	Cohen’s d Value
FIM—motor discharge	44 (26)	27 (14)	0.011	0.640	NS	45 (26)	39 (26)	>0.05	0.233
FIM—motor gain	23 (14)	14 (13)	>0.2	0.424		23 (20)	20 (21)	>0.1	0.179
FIM—cognitive discharge	18 (7)	16.5 (8.7)	>0.6	0.182		18 (7)	17 (8)	>0.5	0.088
FIM—cognitive gain	6.6 (6.0)	9.5 (8.2)	>0.1	−0.476		6.5 (5.7)	7.1 (6.9)	>0.4	−0.095
FIM—total discharge	62 (32)	44 (21)	0.048	0.562	NS	63 (31)	57 (33)	>0.1	0.210
FIM—total gain	29 (24)	23 (20)	>0.5	0.235		30 (23)	27 (26)	>0.3	0.122
DLOTCA—total discharge	83 (36)	56 (27)	>0.1	0.769		81 (35)	85 (38)	>0.5	−0.121
DLOTCA -total gain	26 (24)	7.2 (18.1)	>0.1	0.783		26 (23)	23 (25)	>0.5	0.130
6MW discharge	374 (189)	None	NA ^e^	NA ^e^		367 (174)	392 (224)	>0.5	−0.137
6MW difference ^d^	126 (114)	None	NA ^e^	NA ^e^		120 (111)	138 (121)	>0.6	−0.154

^a^ Outcome when the complication was absent. ^b^ Outcome when the complication was present. ^c^ For differences between presence and absence of the complication. ^d^ Difference = difference between the 6MW distance at discharge from FBR and at start walking without assistance. ^e^ The number of patients in the group was too small for meaningful statistical significance.

**Table 12:** Relationship between procedures at ICCR and outcomes.

Outcome	Cranioplasty					V-P Shunt Insertion			
	No ^a^ Mean (SD)	Yes ^b^ Mean (SD)	*p* ^c^	Cohen’s d value	*p* (Bonf.)	No ^a^ Mean (SD)	Yes ^b^ Mean (SD)	Cohen’s d Value	*p* ^c^
FIM—motor discharge	45 (26)	35 (23)	0.012	0.407	NS	44 (26)	37 (25)	0.262	>0.1
FIM—motor gain	23 (21)	17 (19)	0.039	0.332	NS	23 (21)	18 (19)	0.255	>0.1
FIM—cognitive discharge	18 (8)	15 (6)	0.012	0.405	NS	18 (8)	16 (7)	0.253	>0.1
FIM—cognitive gain	6.8 (6.2)	6.1 (5.7)	>0.4	0.128		6.6 (6.1)	7.1 (5.8)	−0.068	>0.6
FIM—total discharge	64 (32)	50 (27)	0.008	0.427	NS	62 (32)	54 (31)	0.272	>0.1
FIM—total gain	30 (25)	23 (21)	>0.05	0.309		29 (24)	25 (21)	0.194	>0.2
DLOTCA—total discharge	86 (36)	67 (30)	0.035	0.524	NS	82 (36)	88 (34)	−0.193	0.5
DLOTCA—total gain	25 (24)	26 (23)	>0.8	−0.038		24 (24)	32 (22)	−0.370	>0.2
6MW discharge	368 (180)	405 (239)	>0.5	−0.189		376 (188)	359 (207)	0.091	>0.8
6MW difference ^d^	128 (115)	116 (112)	>0.8	0.098		130 (116)	102 (107)	0.244	>0.5

Abbreviations: V-P = ventriculoperitoneal; ^a^ Outcome when the procedure was not performed. ^b^ Outcome when the complication was performed. ^c^ For performance vs. non-performance of the procedure. ^d^ Difference = difference between the 6MW distance at discharge from FBR and at admission.

**Table 13:** Factors associated with outcomes at discharge from FBR (multiple regression).

Outcome	Factor	B ^a^ (95% CI)	95% CI ^boot^	N	R^2 b^	*p* Value	VIF
FIM motor				236	0.671		
	Age	−0.21 (−0.33 to −0.09)	−0.32 to −0.10			0.001	1.203
	Traumatic etiology	13.1 (8.6 to 17.2)	8.5 to 17.5			<0.001	1.270
	Time to admission to ICCR (days)	−2.3 (−4.0 to −0.5)	−4.4 to −0.02			0.013	1.188
	LOS in ICCR (days)	−3.4 (−4.8 to −2.0)	−5.0 to −2.2			<0.001	1.360
	Admission to FBR FIM total	0.66 (0.50 to 0.82)	0.50 to 0.82			<0.001	1.655
FIM cognitive				236	0.463		
	Age	−0.085(−0.129 to −0.042)	−0.130 to −0.041			<0.001	1.202
	Traumatic etiology	3.03 (1.3 to 4.7)	1.4 to 4.7			<0.001	1.269
	LOS in ICCR (days)	−0.91 (−1.42 to −0.40)	−1.51 to −0.44			0.001	1.334
	Admission to FBR FIM total	0.12 (0.06 to 0.18)	0.06 to 0.19			<0.001	1.648
	Hydrocephalus	−1.85 (−3.57 to −0.12)	−3.367 to −0.1			0.036	1.117
FIM total				236	0.678		
	Age	−0.29 (−0.44 to −0.15)	−0.44 to −0.16			0.001	1.203
	Traumatic etiology	16.1 (10.6 to 21.8)	11.0 to 21.5			<0.001	1.270
	Time to admission to ICCR (days)	−2.8 (−5.0 to −0.7)	−5.7 to −0.1			0.011	1.188
	LOS in ICCR (days)	−4.4 (−6.1 to −2.6)	−6.2 to −2.7			<0.001	1.360
	Admission to FBR FIM total	0.78 (0.58 to 0.97)	0.58 to 0.99			<0.001	1.655
	Hydrocephalus	−6.5 (−12.2 to −0.8)	−12.1 to −1.0			0.026	
DLOTCA				86	0.649		
	Admission to FBR DLOTCA total	0.80 (0.60 to 1.0)	0.63 to 0.98			<0.001	1.671

Abbreviations: CI = confidence interval. Boot = estimates after employment of bootstrapping. VIF = variance inflation factor. ^a^ Coefficient (of the variable); <0 means that the factor is negatively associated with the outcome, and >0 means a positive relationship. ^b^ Correlation of determination, squared.

**Table 14:** Factors associated with FBR outcome gain (multiple regression).

Outcome	Factor	B ^a^ (95% CI)	95% CI ^boot^	N	R^2 b^	*p* Value	VIF
FIM motor				236	0.445		
	Age	−0.23 (−0.35 to −0.11)	−0.35 to −0.11			<0.001	1.203
	Traumatic etiology	13.6 (9.0 to 18.2)	9.2 to 18.0			<0.001	1.270
	Time to admission to ICCR (days)	−2.2 (−4.0 to −0.4)	−4.5 to 0.0			0.015	1.188
	LOS in ICCR (days)	−3.5(−4.9 to −2.1)	−5.0 to −2.2			<0.001	1.360
	Hydrocephalus	−5.0 (−9.7 to −0.2)	−9.6 to −0.8			0.040	1.117
FIM cognitive				257	0.128		
	LOS in ICCR (days)	−0.96 (−1.41 to −0.50)	−1.46 to −0.55			<0.001	1.070
	LOS in FBR (days)	0.53 (0.28 to 0.78)	0.29 to 0.92			<0.001	1.166
FIM total				236	0.416		
	Age	−0.29 (−0.44 to −0.15)	−0.43 to −0.15			<0.001	1.203
	Traumatic etiology	16.2 (10.6 to 21.8)	11.0 to 21.9			<0.001	1.270
	Time to admission to ICCR (days)	−2.9 (−5.0 to −0.66)	−5.5 to −0.10			0.011	1.188
	LOS in ICCR (days)	−4.4 (−6.1 to −2.6)	−6.4 to −2.7			<0.001	2.749
	Admission to FBR FIM total	−0.23 (−0.42 to −0.03)	−0.41 to −0.01			0.044	1.655
	Hydrocephalus	−6.5 (−12.2 to −0.8)	−11.8 to −1.1			0.024	1.117
DLOTCA				97	0.102		
	LOS in FBR (days)	2.9	1.0 to 4.6			0.001	Not relevant

^a^ Coefficient (of the variable); <0 means that the factor is negatively associated with the outcome, and >0 means a positive relationship. ^b^ Correlation of determination, squared.

## Data Availability

The data that support this study will be shared onrequest sent to the corresponding author.
